# High-Throughput Sequence Analysis of Peripheral T-Cell Lymphomas Indicates Subtype-Specific Viral Gene Expression Patterns and Immune Cell Microenvironments

**DOI:** 10.1128/mSphere.00248-19

**Published:** 2019-07-10

**Authors:** Hani Nakhoul, Zhen Lin, Xia Wang, Claire Roberts, Yan Dong, Erik Flemington

**Affiliations:** a Department of Pathology, Tulane Cancer Center, Tulane University School of Medicine, New Orleans, Louisiana, USA; b Department of Structural and Cellular Biology, Tulane Cancer Center, Tulane University School of Medicine, New Orleans, Louisiana, USA; University of North Carolina, Chapel Hill

**Keywords:** B-cell receptor diversity, EBV, Epstein-Barr virus, Kaposi's sarcoma-associated herpesvirus, T cell, human T-cell leukemia virus, immune microenvironment, lymphoma, murine leukemia virus, sequencing

## Abstract

Certain peripheral T-cell lymphomas (PTCLs) have been associated with viral infection, particularly infection with Epstein-Barr virus (EBV). However, a comprehensive virome analysis across PTCLs has not previously been reported. Here we utilized published whole-transcriptome RNA sequencing (RNA-seq) data sets from seven different PTCL studies and new RNA-seq data from our laboratory to screen for virus association, to analyze viral gene expression, and to assess B- and T-cell receptor diversity paradigms across PTCL subtypes. In addition to identifying EBV in angioimmunoblastic T-cell lymphoma (AITL) and extranodal NK/T-cell lymphoma (ENKTL), two PTCL subtypes with well-established EBV associations, we also detected EBV in several cases of anaplastic large-cell lymphoma (ALCL), and we found evidence of infection by the oncogenic viruses Kaposi’s sarcoma-associated herpesvirus and human T-cell leukemia virus type 1 in isolated PTCL cases. In AITLs, EBV gene expression analysis showed expression of immediate early, early, and late lytic genes, suggesting either low-level lytic gene expression or productive infection in a subset of EBV-infected B-lymphocyte stromal cells. Deconvolution of immune cell subpopulations demonstrated a greater B-cell signal in AITLs than in other PTCL subtypes, consistent with a larger role for B-cell support in the pathogenesis of AITL. Reconstructed T-cell receptor (TCR) and B-cell receptor (BCR) repertoires demonstrated increased BCR diversity in AITLs, consistent with a possible EBV-driven polyclonal response. These findings indicate potential alternative roles for EBV in PTCLs, in addition to the canonical oncogenic mechanisms associated with EBV latent infection. Our findings also suggest the involvement of other viruses in PTCL pathogenesis and demonstrate immunological alterations associated with these cancers.

**IMPORTANCE** In this study, we utilized next-generation sequencing data from 7 different studies of peripheral T-cell lymphoma (PTCL) patient samples to globally assess viral associations, provide insights into the contributions of EBV gene expression to the tumor phenotype, and assess the unique roles of EBV in modulating the immune cell tumor microenvironment. These studies revealed potential roles for EBV replication genes in some PTCL subtypes, the possible role of additional human tumor viruses in rare cases of PTCLs, and a role for EBV in providing a unique immune microenvironmental niche in one subtype of PTCLs. Together, these studies provide new insights into the understudied role of tumor viruses in PTCLs.

## Abstract (precis)

In this study, we utilized next-generation sequencing data from 7 different studies of peripheral T-cell lymphoma (PTCL) patient samples to globally assess viral associations, provide insights into the contributions of EBV gene expression to the tumor phenotype, and assess the unique roles of EBV in modulating the immune cell tumor microenvironment. These studies revealed potential roles for EBV replication genes in some PTCL subtypes, the possible role of additional human tumor viruses in rare cases of PTCLs, and a role for EBV in providing a unique immune microenvironmental niche in one subtype of PTCLs. Together, these studies provide new insights into the understudied role of tumor viruses in PTCLs.

## INTRODUCTION

Peripheral T-cell lymphomas (PTCLs) are relatively uncommon malignancies, comprising less than 15% of non-Hodgkin's lymphomas worldwide. However, their heterogeneity of presentation, aggressive clinical course, and poor prognosis present serious challenges to pathologists and clinicians (1). Recent genomic and transcriptomic studies have led to rapid changes in the molecular classification of PTCLs, identifying recurrent driver mutations and highlighting common pathogenic mechanisms (reviewed in references [Bibr B2][Bibr B3][Bibr B4]). These mechanisms include derangements of T-cell receptor signaling and other oncogenic pathways in neoplastic cells, stimulatory signaling by the tumor microenvironment, and pathogen-mediated immunoevasive, proproliferative, and antiapoptotic effects (4, 5).

Epstein-Barr virus (EBV) is a human gammaherpesvirus that plays an etiological role in the pathogenesis of several malignancies, including lymphoma, gastric carcinoma, and nasopharyngeal carcinoma (6). The oncogenic roles of EBV in B-cell lymphomas are particularly well established, but consistent associations between EBV infection and certain PTCL subtypes have also been observed (7, 8). In extranodal NK/T-cell lymphoma (ENKTL), EBV directly infects tumor cells of T- or NK-cell lineage and is consistently detected in episomal form in these cells. On the other hand, in angioimmunoblastic T-cell lymphoma (AITL), EBV-infected B cells are found adjacent to tumor cells of the T follicular helper (Tfh) phenotype. It has been proposed that interaction between the tumor cells and the B lymphocytes provides oncogenic support for the tumor, although the mechanism for this process is not clear. In still other PTCL subtypes, including anaplastic large-cell lymphoma (ALCL) and peripheral T-cell lymphoma not otherwise specified (PTCL-NOS), reports of cases involving EBV and other viruses have been limited or controversial. Studies of EBV in NK- and T-cell neoplasms have been complicated by a relative lack of cell lines and animal models that replicate key features of these diseases. Although some high-throughput sequencing studies have confirmed the presence of EBV in particular PTCL subtypes, a comprehensive virome analysis across PTCL subtypes has not, to our knowledge, been previously reported.

Latent EBV infection is associated with the constitutive expression of a subset of viral genes without the production of infectious virions. The particular viral genes expressed, which depend on immune surveillance and the characteristics of the host cell, define the latency type of the EBV infection (9). EBV-associated cancers are each typically associated with a specific latency type, although even within a tumor type, patient-to-patient and intratumoral variations exist. Endemic Burkitt's lymphoma typically exhibits latency type I, in which EBV expresses only the episomal maintenance factor EBNA1, the noncoding RNAs EBER1 and EBER2 (10), the BART long noncoding RNAs (lncRNAs) (referred to here as RPMS1 and A73) (11–14), the BART microRNAs (15–18), and the circular RNAs circRPMS1_E4_E3a and circRPMS1_E4_E2 (19–24). Other lymphomas in immunocompetent patients, gastric carcinoma, and nasopharyngeal carcinoma usually exhibit type II latency, in which LMP1 and LMP2A are expressed, in addition to type I genes (25, 26). Lymphomas in the setting of immunosuppression often exhibit type III latency, in which all EBV latent genes are expressed (27, 28). The lytic or productive phase of EBV infection is not classically associated with malignancy, although lytic cycle proteins have been shown to play a role in the pathogenesis of certain tumors (29–32).

Both lytic and latent EBV infections are subject to surveillance by the host immune system through antigen-specific T- and B-cell responses. EBV-associated cancers in immunocompetent patients demonstrate a variety of strategies for immune evasion, including suppression of antigen presentation through downregulation of the major histocompatibility complex (MHC) and upregulation of the immunomodulatory host genes PD-L1 and IDO1 (6). In the case of lymphoid malignancies, immune dysfunction is also caused by derangements of B-cell receptor (BCR) and T-cell receptor (TCR) signaling in neoplastic cells themselves (33, 34). Functional studies of BCR and TCR repertoires and their interactions with abnormalities in particular cancers are now possible due to both efficient high-throughput methods for targeted amplification of BCRs and TCRs and computational techniques that permit reconstruction of these repertoires from untargeted whole-transcriptome sequencing data (35–37).

Here we make use of whole-transcriptome RNA sequencing (RNA-seq) data from seven previously published studies of PTCL primary tumors and cell lines (38–44), as well as cell line RNA-seq data from our own laboratory, to broadly screen for virus association, investigate viral gene expression profiles, and determine the properties of reconstructed TCR and BCR repertoires. In agreement with previous studies (45–48), we found consistent evidence of EBV in AITLs and ENKTLs. In addition, we detected EBV in several cases of ALCL, two cases of which displayed high levels of EBV expression. We found isolated cases of primary tumors that were positive for the oncogenic viruses Kaposi’s sarcoma-associated herpesvirus (KSHV) and human T-cell leukemia virus type 1 (HTLV-1), raising the possibility that these are sporadically involved in the pathogenesis of T-cell malignancies. In AITLs, we observed the expected EBV latency gene expression profile but also substantial expression of lytic genes, representing evidence of abortive lytic replication and/or productive replication in a subset of infected cells. Deconvolution of immune cell subpopulations from RNA-seq data showed greater B-cell signals and increased BCR repertoire diversity in AITLs, consistent with a possible EBV-driven polyclonal response.

## RESULTS

### Virus read detection.

EBV contributes to the etiology of a number of PTCL subtypes (1, 7, 49) with a high prevalence in AITLs (50) and ENKTLs (51). While the cellular transcriptomes of AITL, ALCL, ENKTL, and PTCL-NOS patient samples have been previously analyzed by RNA sequencing (38–43), the viral compositions of these transcriptomes were not considered in these analyses. As potential drivers of tumor progression in PTCLs, the viral contributions to the overall transcriptomes are germane to understanding the mechanisms driving the tumor phenotype.

Our initial approach to assessing the viral impact on these tumors was to take a macroscopic view of viral etiology by analyzing previously published clinical data sets (38–43) for reads mapping to a panel of mammalian viruses (52, 53). Reads from each sample were aligned to a genome index containing all human chromosomes plus 740 genomes for viruses known to infect human cells (listed in Table S1 in the supplemental material), as we have done for previous studies (52, 53). To limit false-positive calls related to contamination (54) or low-level virus presence in a small percentage of stromal cells, we utilized a threshold frequency of 0.2 read per million mapped human reads (RPMHRs). This cutoff is in rough agreement with that used in previous investigations by our group and others assessing exogenous viruses in sequencing data (52, 55–57).

10.1128/mSphere.00248-19.4 TABLE S1 Viral genomes assessed in this study. Accession numbers are from NCBI GenBank, unless otherwise specified. Download Table S1, XLSX file, 0.1 MB. Copyright © 2019 Nakhoul et al. 2019 Nakhoul et al. This content is distributed under the terms of the Creative Commons Attribution 4.0 International license.

**FIG 1:**
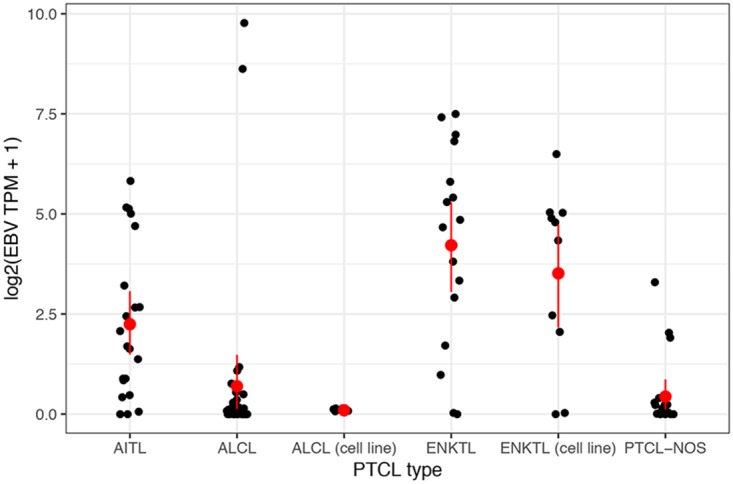
Distribution of total EBV gene expression (transcripts per million [TPM]) for each PTCL subtype. For each sample, the *y* axis indicates the sum of TPMs of all EBV genes. Red bars indicate 95% bootstrap confidence intervals about the mean.

**FIG 2:**
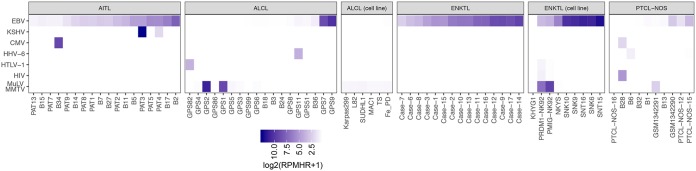
Viruses detected in RNA-seq data from PTCLs. Only those samples with at least 0.2 read per million human mapped reads (RPMHR) are shown.

In line with previous reports (50, 51), EBV was the most prevalent virus detected across all PTCL subtypes, with 18 of 21 AITL and 15 of 17 ENKTL patient samples being positive for EBV (Fig. 1 and 2). While infiltrating EBV-positive B lymphocytes are characteristic of AITL tumors (reviewed in reference 58), the etiology of ENKTLs involves direct EBV infection of the tumor cells of NK- or T-cell lineage (40, 59). Consistent with direct infection of ENKTL tumor cells, 8 of 10 ENKTL cell lines that we analyzed, including 5 sequenced by our lab, were found to be positive for EBV. Eleven of 35 ALCL samples and 7 of 21 PTCL-NOS samples were also positive for EBV, although only 2 ALCLs showed high levels of EBV reads (451 and 1,158 RPMHRs) and only 3 PTCL-NOS samples showed at least moderate levels of EBV reads (2, 6, and 9 RPMHRs). These results are consistent with the established role of EBV in the pathogenesis of AITL and ENKTL, while at the same time they raise the possibility of the involvement of EBV in a subset of ALCL and PTCL-NOS cases (60–62).

**FIG 3:**
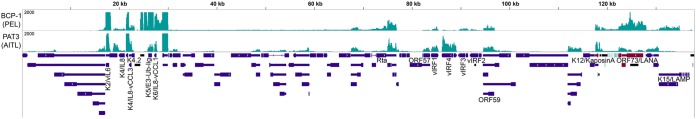
KSHV exhibits an expression profile consistent with latent infection in one AITL sample (PAT3). KSHV coverage is also shown for BCP-1, a cell line derived from a primary effusion lymphoma. Transcripts were detected from the primary latency transcript region located between the K12 and ORF74 open reading frames, K2/viral IL-6 (vIL-6), the noncoding RNA PAN, and the viral IRF (vIRF) family of transcription factors.

In addition to pervasive evidence of EBV in these PTCL patient and cell line samples, one AITL patient sample contained high read numbers (5,767 RPMHRs) for another oncogenic gammaherpesvirus, KSHV, with a second AITL sample containing lower but potentially meaningful numbers of KSHV reads (3 RPMHRs) (Fig. 2). Viral transcriptome coverage from the AITL sample with high KSHV detection was similar to that observed from the KSHV-positive primary effusion lymphoma cell line BCP-1, with expression of the classic latency genes LANA and Kaposin (63, 64), as well as expression of the viral interleukin-6 (IL-6) and IL-8 homologues (65, 66) and the viral E3 ubiquitin ligase (67) (Fig. 3). These findings indicate a predominantly latent infection that may contribute to the tumor phenotype in these patients.

Although betaherpesviruses, such as the ubiquitous human cytomegalovirus (CMV) and the human herpesvirus 6 (HHV-6), are not classically considered tumor viruses, there are a number of sometimes controversial reports of their unconventional roles in cancer (68–71). Whether it is etiological or detected due to local immune suppression in the tumor microenvironment, we have previously detected HHV-6 in occasional B-cell lymphomas (53). Here, we similarly detected moderate to low levels of HHV-6 in an ALCL and a PTCL-NOS, and we detected high levels of CMV in one AITL and lower levels in a PTCL-NOS (Fig. 2 and Fig. S1). As we suggested for our previous HHV-6 B-cell lymphoma findings, the results here could simply represent tolerance for replication of these ubiquitous viruses in a local immune-suppressed microenvironment.

10.1128/mSphere.00248-19.1 FIG S1 Genomic coverage of CMV (A) and HIV (B) in B28 (PTCL-NOS), B34 (AITL), and B36 (ALCL) samples. Download FIG S1, PDF file, 1.5 MB. Copyright © 2019 Nakhoul et al. 2019 Nakhoul et al. This content is distributed under the terms of the Creative Commons Attribution 4.0 International license.

HIV reads were detected at low levels in one ENKTL cell line and one PTCL-NOS patient (Fig. 2 and Fig. S1). With little previous evidence of a direct oncogenic role of HIV, similar to the findings of betaherpesviruses in some tumor samples, this could represent replication in an immune-compromised microenvironment in the PTCL-NOS sample and possible carryover or tissue culture contamination with HIV in the ENKTL cell line. On the other hand, HTLV-1 is a known oncogenic T-cell tumor virus, and the detection of HTLV-1 in one ALCL (Fig. 2) could represent a pathological infection.

Lastly, the murine leukemia virus (MuLV) and its relative, the mouse mammary tumor virus (MMTV), were detected in two ENKTL cell lines (Fig. 2). While initially thought to be pathological infections in prostate cancers, cell line findings for MuLV were later found to be due to propagation of the respective cell lines in MuLV-infected mice, where they picked up the virus (72, 73). The presence of MuLV/MMTV reads in multiple cell lines is consistent with cross-contamination or infection during culture (74). What is particularly striking here, however, is the unusually high levels of MuLV/MMTV in two ALCL patient samples (Fig. 2). We investigated the metadata for this study and could not identify any potential artifactual evidence to explain these findings. Clearly, these murine viruses can infect human cells, and there is the formal possibility that MuLV could play an etiological role in these patients. However, given the pervasiveness of MuLV in laboratories and previous erroneous reports, such conclusions must be made in an extremely guarded fashion.

### EBV gene expression analysis.

**FIG 4:**
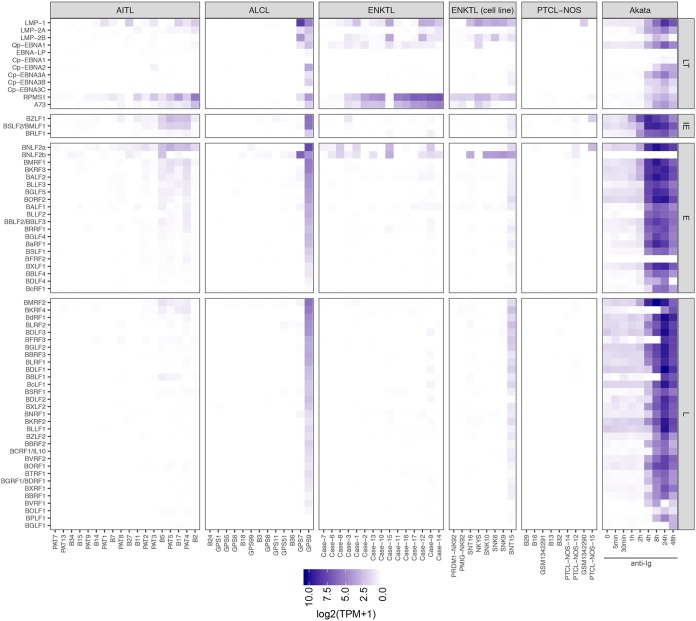
Expression of latent (LT), immediate early lytic (IE), early lytic (E), and late lytic (L) EBV genes in PTCL primary tumors and cell lines. Columns indicate PTCL samples, and each row indicates an EBV gene. In the rightmost panel, expression data from the EBV-positive Burkitt lymphoma cell line Akata is shown at several time points, as lytic reactivation was induced with human IgG.

The results of our screen for viruses in PTCLs confirm EBV as the viral pathogen most consistently associated with PTCLs. EBV gene expression patterns vary across malignancies and, to some degree, between patients with a specific tumor type. The unique pattern of viral genes expressed in a particular tumor setting is directly pertinent to viral oncogenesis in the respective tumor type and/or patient (9, 75). Although EBV latency types have been previously defined in AITL and ENKTL on the basis of immunohistochemistry, comprehensive sequencing-based analyses of EBV gene expression have not been performed to date for AITL and have only recently been performed for ENKTL (76). We therefore studied the distribution and variation of EBV gene expression across PTCL subtypes. Figure 4 shows the expression of individual EBV genes in EBV-positive PTCL samples, as well as in the EBV-positive Burkitt lymphoma-derived Akata cell line, before and after induction of the lytic cycle with anti-human IgG (77, 78). In AITLs, where EBV is present in the stromal B cells, variable expression of LMP1 and LMP2A/B was detected, consistent with previous reports of type II latency infection in AITLs (60, 79) (Fig. 4 and Fig. S2). More consistently expressed were the EBV noncoding BART lncRNAs RPMS1 and A73, whose expression *in vivo* can exert effector signaling to the host cell regulatory circuitry without eliciting an adaptive immune response. Also notable is detection of immediate early BZLF1 and BRLF1 transcripts as well as some early and late lytic genes in AITLs but not ENKTL patient or cell lines (Fig. 4). While this may represent a small percentage of cells undergoing lytic replication, their expression levels rival those of the latency genes (Fig. 4). These results are consistent with previous reports detecting expression of the immediate early Zta gene by immunohistochemistry in AITL patients (80). They are also consistent with our findings of relatively high lytic gene expression in B-cell lymphoma patient samples (53), suggesting the common observation of lytic gene expression in B cells *in vivo*. Since early genes, such as BZLF1, are expressed transiently during initial infection of naive B cells (81, 82), where they provide an initial growth stimulation (83), BZLF1 and other lytic genes may similarly support the proliferation of the stromal B-cell population in AITLs.

10.1128/mSphere.00248-19.2 FIG S2 Expression of EBV latency genes for several selected PTCL types, indicating variable expression of LMP1 and LMP2 in AITL, ALCL, and ENKTL. Download FIG S2, PDF file, 0.01 MB. Copyright © 2019 Nakhoul et al. 2019 Nakhoul et al. This content is distributed under the terms of the Creative Commons Attribution 4.0 International license.

The antigenic type III latency EBNA proteins were generally not detected in AITLs, with the exception of EBNA2 expression in patient 3 (Fig. 4 and Fig. S2). EBNA2 expression in this patient coincides with KSHV infection. In the setting of EBV and KSHV coinfection, EBV has recently been shown to interact with KSHV and enhance the stability of KSHV infection (84). However, whether KSHV regulates EBNA2 expression in this case, whether this tumor displays generally higher local immune suppression to tolerate two viral infections as well as EBNA2 expression, or whether this is a coincidental observation remains to be determined.

In ENKTLs, we similarly observed primarily evidence of type II latency, consistent with previous studies (9), and robust expression of the BART lncRNAs RPMS1 and A73 (Fig. 4 and Fig. S2). ALCLs generally exhibited very low expression of EBV genes in most EBV-positive samples. However, 2 of 15 EBV-positive samples showed high levels of EBV gene expression, and 1 of these showed high levels of lytic gene expression (Fig. 4).

### Extensive B-cell receptor repertoire diversity in AITLs.

**FIG 5:**
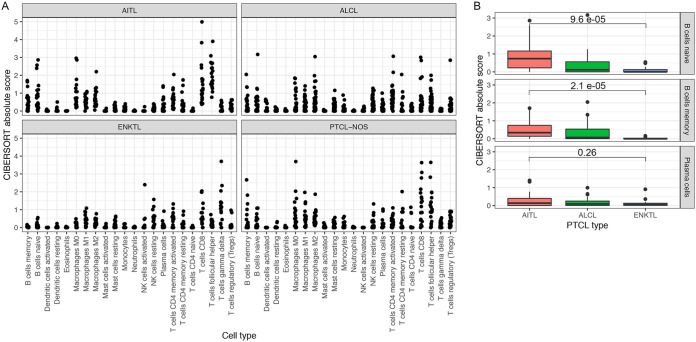
(A) Enrichment scores for 22 immune cell subpopulations based on deconvolution by CIBERSORT. Each panel indicates a subtype of primary tumor. (B) Box plots of enrichment scores for three B-cell subpopulations in AITL, ALCL, and ENKTL primary tumor samples. For naive and memory B cells, scores are significantly greater in AITLs than in ENKTLs.

In most EBV-associated tumors, EBV is present within the tumor cell, where the expressed viral genes contribute directly to the tumor phenotype. AITLs are unusual in that EBV infects the stromal B cells, which appear to provide essential support to the T-cell tumor (reviewed in reference 7). To explore the tumor and stromal cell composition across these PTCL subtypes, we performed deconvolution of gene expression profiles to estimate the immune cell composition in each tumor sample (85) (Fig. 5A). Consistent with the proposed requirement of a tumor-supporting B-cell population in AITLs, there is comparatively high B-cell evidence in AITLs relative to ALCLs and ENKTLs, with ENKTLs showing low B-cell signals and ALCLs showing intermediate B-cell signals (Fig. 5B). These results also indicated enrichment of the T follicular helper subtype of immune cells in AITLs, consistent with the characterization of AITL itself as a neoplasm of the T follicular helper phenotype (1, 86–88). This analysis also showed enrichment of other immune cell subpopulations, such as macrophages, consistent with the known heterogeneous tumor microenvironment of AITL (89). As an indication of the validity of the deconvolution method, enrichment of B cells and macrophages was not detected in cell lines and purified T-cell samples (Fig. S3). B-cell and macrophage scores were decreased in ENKTL relative to AITL. These results indicate that the pathological characterization of the heterogeneity of the tumor microenvironment in PTCLs is broadly reproducible from RNA-seq data.

10.1128/mSphere.00248-19.3 FIG S3 Enrichment scores for 22 immune cell subpopulations in cell lines based on deconvolution by CIBERSORT. Each panel indicates a subtype of a cell line or a purified population of immune cells. Download FIG S3, PDF file, 0.04 MB. Copyright © 2019 Nakhoul et al. 2019 Nakhoul et al. This content is distributed under the terms of the Creative Commons Attribution 4.0 International license.

### T- and B-cell receptor analyses.

**FIG 6:**
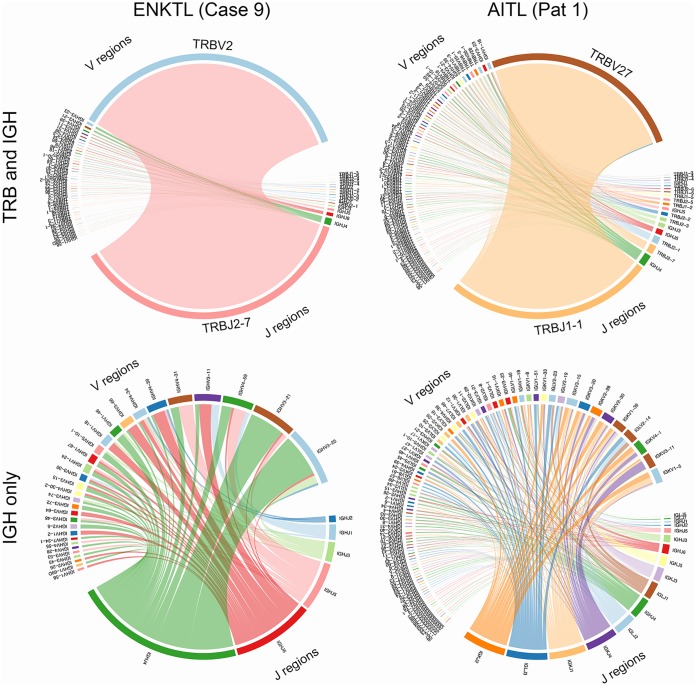
Circle plots indicating V(D)J recombination in an AITL sample and an ENKTL sample.

**FIG 7:**
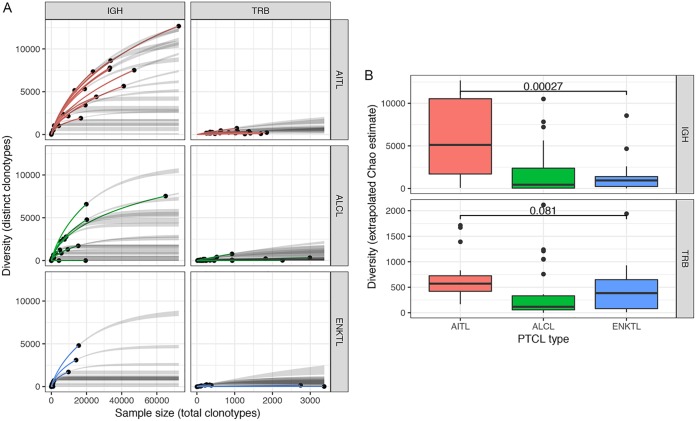
(A) Rarefaction plots for AITL, ALCL, and ENKTL samples, based on multinomial models of diversity as a function of sample size (91, 128). The *x* axis indicates the number of clonotypes detected in each sample (sample size), and the *y* axis indicates the number of distinct clonotypes (diversity). Solid dots indicate the observed sample size and diversity, colored lines indicate interpolation of a curve to this value, and gray lines indicate extrapolation of the curve to the largest sample size in the data set. (B) Box plots showing extrapolated Chao estimates, a diversity measure that permits comparisons of clonotype diversity across samples with distinct numbers of total clonotypes (91). IGH diversity is significantly greater in AITLs than in ENKTLs (Wilcoxon rank-sum test).

Since enrichment of EBV-positive B lymphocytes in the tumor microenvironment is characteristic of AITLs (45, 46, 90), we hypothesized that T-cell receptor (TCR) and B-cell receptor (BCR) repertoire diversity would vary across PTCL subtypes, with greater BCR diversity in AITLs due to polyclonal EBV-mediated B-cell expansion. To test this hypothesis, we reconstructed BCR sequences from the RNA-seq data and assessed the relationship between total assembled clonotypes and unique clonotypes. We first assessed this by constructing circle plots displaying TCR (T-cell receptor β [TRB]) and BCR (immunoglobulin heavy chain [IGH]) V(D)J recombinations for each AITL and ENKTL. This analysis showed considerable variation across samples but generally displayed monoclonal TCR expansions likely of tumor cell origin, with typically lower T- and B-cell subpopulations that likely represent infiltrating immune cells (representative plots are shown in Fig. 6, top; note the major TRB J2-7 joining region-to-V2 variable region clone in the ENKTL patient and the major TRB J1-1 joining region-to-V27 variable region clone in the AITL patient). Assessing the BCR repertoire alone, variation was observed across samples, but in general, greater diversity was seen in AITLs (a representative plot is shown in Fig. , bottom; note the higher number of J regions and the greater number of V region subclones in the AITL sample). This greater B-cell diversity in AITLs may be driven by EBV-mediated polyclonal expansion. To investigate the relationship between the size of the reconstructed BCR repertoires and their diversity, we generated rarefaction plots, an approach borrowed from the ecological literature that is now commonly used in computational immunology (35–37). We computed extrapolated Chao estimates of the diversity of B- and T-cell receptor clonotypes in each sample, permitting comparison of these repertoires across different sampling depths (91). Using this approach for the AITL, ALCL, and ENKTL PTCL subtypes, a greater number of total and unique BCR clonotypes were observed in AITLs than in ALCLs or ENKTLs, indicating a greater average BCR diversity for AITLs (Fig. 7A and B). This suggests that EBV facilitates polyclonal B-cell expansion in AITLs, whereas in infiltrating B cells in ALCLs and ENKTLs, less diversity is observed due to expansion of a relatively small number of antigen-reactive clones.

## DISCUSSION

### Frequent detection of viruses in PTCL samples.

Viruses exert characteristic effects on cancer progression and define unique subtypes of malignancies (28, 92). Although the association between EBV and certain PTCL subtypes has been firmly established on the basis of immunochemistry, our study is, to our knowledge, the first unbiased virome analysis across PTCL subtypes. The sporadic detection of oncogenic viruses, such as KSHV and HTLV-1, in this study raises the possibility that, in addition to EBV, these viruses may similarly contribute to the pathogenesis of PTCL in rare cases.

KSHV infection is critical to the pathogenesis of several lymphoproliferative disorders, including multicentric Castleman disease, immunodeficiency-related diffuse large B-cell lymphoma, and primary effusion lymphoma (93). These disorders are heterogeneous in clinical presentation, histology, immunophenotype, and EBV involvement; for example, cases of primary effusion lymphoma with the T-cell phenotype have been reported, although an indeterminate immunophenotype is more common (94). There have been previous reports of occasional KSHV-positive cases of AITL, as determined by PCR (95), although this finding was not reproduced in subsequent studies by either PCR or immunohistochemistry (96–98). The quantitative nature of our approach revealed a strong presence of KSHV in 1 of 22 AITLs, supporting an etiological role for KSHV in a minor percentage of cases of this tumor type. Although rare in the AITL cases assessed here, it will be important to determine whether KSHV displays a more substantial penetrance in areas of high KSHV seroprevalence (e.g., sub-Saharan Africa) (99).

In contrast to previous studies that reported the detection of HHV-6 in 20 to 50% of AITL samples by PCR (98, 100) and an association between HHV-6 copy number and histological progression (96), we did not find consistent evidence of HHV-6 in any of the RNA-seq cohorts analyzed here. This negative finding should be considered in the context of the pervasive detection of EBV transcripts among these AITL samples, which serves as a reference for detection of viruses in these samples. Further, with EBV localized within the stromal B cells rather than the tumor cells, this further emphasizes the discrepancy in EBV and HHV-6 detection. This suggests that perhaps the HHV-6 detected by PCR would likely be confined to a minor fraction of stromal and/or tumor cells.

### EBV in AITL.

A unique feature of AITL is its heterogeneous tumor microenvironment, marked by the coexistence of neoplastic T cells and (frequently EBV-positive) infiltrating B cells. B-cell proliferation and the subsequent development of B-cell lymphoma are common clinical features of AITL (45, 46, 60, 101, 102). Furthermore, recurrent mutations in AITL are cell type specific, with RHOA and IDH2 mutations occurring exclusively in neoplastic PD-1-positive T cells, NOTCH1 mutations occurring exclusively in B cells, and TET2 mutations occurring in both T and B cells (90, 103). EBV is believed to contribute to the neoplastic transformation of B cells in the progression from AITL to B-cell lymphoma, although EBV is not uniformly observed in these cases (104).

Interactions between EBV-infected cells and other cells in the tumor microenvironment, representing a subversion of normal mechanisms of immune signaling, are a common feature in lymphoid malignancies (6) and represent a possible mechanism for immune evasion or modulation in these settings (105). In the case of AITL, previous studies of B-cell receptor repertoires have suggested that EBV supports the survival of clonal expansions of B cells with unfavorable BCR mutations, permitting them to escape B-cell selection (46). We present support for this mechanism in our demonstration of expanded B-cell repertoires in AITL, although current technology precludes a complete study of somatic mutations in BCR repertoires from RNA-seq data (36). In our study, the finding of substantive expression of a subset of lytic EBV genes is consistent with a possible decrease in the immune control of viral infection in AITLs, perhaps allowing for the growth-stimulatory functions of these lytic genes in contributing to tumor cell growth. Indeed, a recent study of chronic active EBV infection, a lymphoproliferative disorder characterized by EBV-infected T/NK cells, has shown recurrent intragenic deletions in the EBV genome that upregulate lytic genes and promote lymphomagenesis in a xenograft model (106). Together, these findings affirm that immune modulation, possibly directly mediated by EBV, is a significant feature of the pathogenesis of AITL.

### EBV in ENKTL.

Although EBV infection is consistently associated with the pathogenesis of ENKTL, the fact that the virus infects neoplastic NK and T cells implicates risk factors and oncogenic pathways that are distinct from those involved in EBV-associated B-cell malignancies (reviewed in references 7 and 8). These include recurrent loss-of-function mutations in the RNA helicase DDX3X, which has been conjectured to play a role in the interaction between viral and cellular proteins (59), and polymorphisms in HLA alleles that play a role in antigen processing to T lymphocytes (107). As in other EBV-associated malignancies, the viral oncogene LMP1 has been shown to promote immune evasion by upregulating PD-L1, which is a poor prognostic factor for ENKTL (108). A recent study has analyzed EBV genomes, transcriptomes, and T-cell epitopes in an independent cohort of ENKTL patients recruited from two centers in China and Singapore (76). Findings from this study include recurrent long-fragment deletions at the viral BART locus, integration of short EBV genomic sequences at the host NHEJ1 gene, and overexpression of lytic genes relative to EBV-associated gastric carcinoma and nasopharyngeal carcinoma. However, in our data set, less lytic expression was observed in ENKTL than in AITL, and expression of the BNLF2a and BNLF2b genes is consistent with the possibility of latent infection, as our group has shown in the case of gastric carcinoma (109).

### EBV in ALCL.

The role of EBV in ALCL has been controversial (reviewed in references 110 and 111). Although early studies claimed to demonstrate EBV genomes and latent gene products in certain ALCL samples (112, 113), a more recent study of 64 cases defined according to the WHO criteria concluded that EBV plays no role in the pathogenesis of ALCL among Western patients (114). On the other hand, rare cases of EBV-positive CD30^+^ ALCLs have been reported in case series of South Korean patients (62), and recent case reports have highlighted cases of EBV-positive ALCLs, particularly in the setting of immunosuppression (115, 116). Our data suggest that low-level EBV expression can occasionally be detected in ALCLs and that these cases likely represent prototypical ubiquitous infection rather than a tumorigenic event. Nevertheless, two samples showing high EBV levels and a lytic expression pattern were found in this cohort, suggesting that EBV might play a more active tumorigenic role in some patients.

### Diversity of T-cell and B-cell repertoires.

Previous studies have reconstructed TCR repertoires from RNA-seq data for PTCLs in order to assess T-cell clonality, differential patterns of V(D)J recombination, and abnormalities in TCR expression, using both multiplex PCR (61) and RNA-seq (43). Although analogous studies of BCR repertoires from RNA-seq are limited by insufficient resolution to detect phenomena such as somatic hypermutation and class switching, it is possible to assess BCR clonality, diversity, and recombination patterns from RNA-seq (36, 37). Although EBV infection has been associated with decreased TCR diversity in some EBV-positive cancers, reflecting a possible antigen-driven proliferative response (117), another study has tied EBV infection to increases in B-cell receptor diversity and abundance, likely due to increased infiltration of B lymphocytes (57). The abnormalities of TCR repertoires that are associated with T-cell neoplasms, as well as an incomplete understanding of CDR3-epitope relationships for EBV, preclude analysis of the functional characteristics of the T-cell response to EBV in PTCLs. However, the increased immunoglobulin heavy chain (IGH) abundance and diversity noted in AITLs relative to other PTCL subtypes is consistent with a proliferative response driven by both the direct effects of EBV and abnormalities of T-cell signaling.

## MATERIALS AND METHODS

### Data sets.

RNA-seq data from PTCL patient tumors and cell lines and from isolated subpopulations of T lymphocytes were obtained from the NCBI Sequence Read Archive (SRA) and Database of Genotypes and Phenotypes (dbGaP) (38–44) (accession numbers SRP029591, SRP040799, SRP049695, SRP044708, SRP039591, SRP099016, and SRP043339). We also performed RNA sequencing of five EBV-positive ENKTL tumor cell lines (SNK6, SNK9, SNK10, SNT15, and SNT16) characterized previously (118, 119). For ENKTL cell line sequencing, total RNA was extracted from cultured cells with the TRIzol reagent (catalog no. 15596-018; Life Technologies) according to the manufacturer’s protocol. Library preparations of polyadenylated RNAs were generated using an Illumina TruSeq stranded mRNA library preparation kit, and 100-base single-end sequencing was performed using an Illumina HiSeq 2000 sequencer (University of Wisconsin Biotechnology Center).

### Virus read detection.

Virus read detection was performed as previously described (52) with minor modifications. Reads were aligned to a reference genome containing the GRCh37 assembly of the human genome and 740 mammalian virus genomes (NCBI) (see Table S1 in the supplemental material) using the short-read aligner STAR (options chimOutType WithinBAM, and outFilterMultimapNmax 50) (120). To reduce the number of false-positive alignments to regions of viral genomes that share homology with the human genome (for example, see reference 121 for human herpesvirus-6 and -7/human telomere homology artifacts), only primary alignments in which both reads of a read pair were properly mapped to the reference were considered hits. Furthermore, reads aligning to five viruses in the panel (Table S2) were judged to be likely of human origin, based on manual assessment, and were excluded from further analysis (52, 53). Specifically, as in previous studies by our group, low numbers of reads aligning to three strains of the hepatitis C virus genome mapped to a poly(T) tract in the viral genome and were considered to be likely derived from poly(A) tracts of cellular mRNAs (52). Shamonda and Simbu orthobunyavirus mapped reads were found to align to short sections of these viral genomes with strong homology to human rRNA, and these reads were similarly assumed to be of human origin. Lastly, some low-level viral read counts were found to be consistent with the possibility of cross-sample contamination or other artifacts (54). For example, adenovirus C reads in one ALCL sample aligned to a portion of the adenovirus genome that was also included in multiple cloning vectors, indicating a possible artifact of library preparation (data not shown).

10.1128/mSphere.00248-19.5 TABLE S2 Viral genomes judged to be likely of human origin, based on manual assessment, that were excluded from further analysis. Download Table S2, TXT file, 0.01 MB. Copyright © 2019 Nakhoul et al. 2019 Nakhoul et al. This content is distributed under the terms of the Creative Commons Attribution 4.0 International license.

### Gene expression analysis.

Gene expression levels based on human (Ensembl v.85) and EBV (77, 122) transcriptomes were determined using the Salmon (v.0.8.2) program run with default parameters (123). The EBV gene expression data from the Akata cell reactivation time course were described previously (77). Deconvolution of immune cell subpopulations from RNA-seq data was performed using the CIBERSORT (v.1.0.6) tool with the default LM22 data set as a signature gene file. Absolute scores, which were estimated as the median expression level of genes in the signature file divided by the median expression level of all genes in the sample, were computed for each subpopulation to permit comparisons across samples (85, 124). Plots of genomic coverage were generated with the Integrated Genomics Viewer (IGV) genome browser (125). Scatter plots, box plots, line plots, and heat maps were generated with the ggplot2 package (v.3.1.0) (126).

### T- and B-cell receptor analyses.

T- and B-cell receptor repertoires were reconstructed from RNA-seq data using the MiXCR (v.3.0.3) program run with default parameters for nontargeted RNA-seq libraries (127). Sequence correction of erroneous clonotypes and filtering of nonfunctional clonotypes were performed using the vdjtools (v.1.2.1) framework (128). Calculation of repertoire diversity, V(D)J segment usage, and sample overlap for immunoglobulin heavy chain (IGH) and T-cell receptor β (TRB) repertoires were also performed with vdjtools. Rarefaction plots were generated for the IGH and TRB repertoires, using multinomial models both for interpolation to the observed diversity and for extrapolation to the largest sample size in the data set (91). The extrapolated Chao diversity estimate, as implemented in vdjtools (128), was used to compare the diversity of the IGH and TRB repertoires across samples of different sizes. Briefly, this is an abundance-based coverage estimator in which frequency counts for rare clonotypes are accounted for in the estimate (see reference 91 for details).

### Data availability.

The RNA sequencing data generated for this study have been submitted to the NCBI GEO repository (accession number GSE131261).
