# Supplementary figures and images for: High-Throughput Sequence Analysis of Peripheral T-Cell Lymphomas Indicates Subtype-Specific Viral Gene Expression Patterns and Immune Cell Microenvironments
Source: mSphere. 2019 Jul 10;4(4):e00248-19. doi: 10.1128/mSphere.00248-19 (PMC6620372; doi:10.1128/mSphere.00248-19)

A

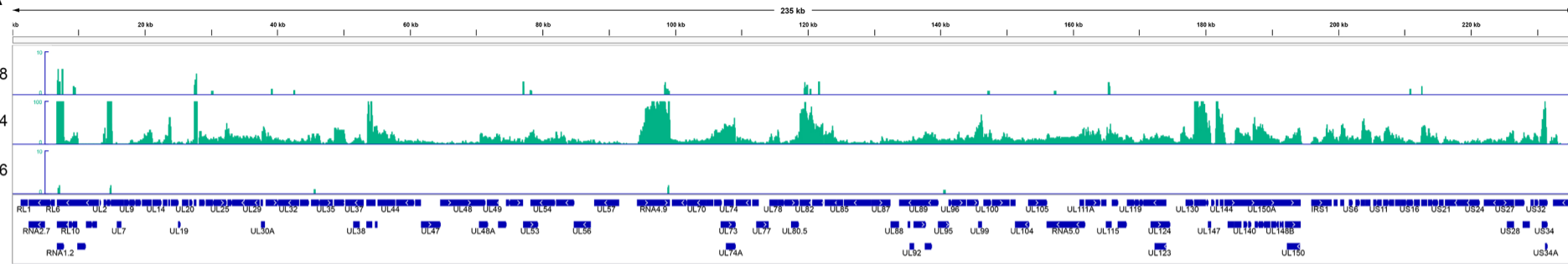

B

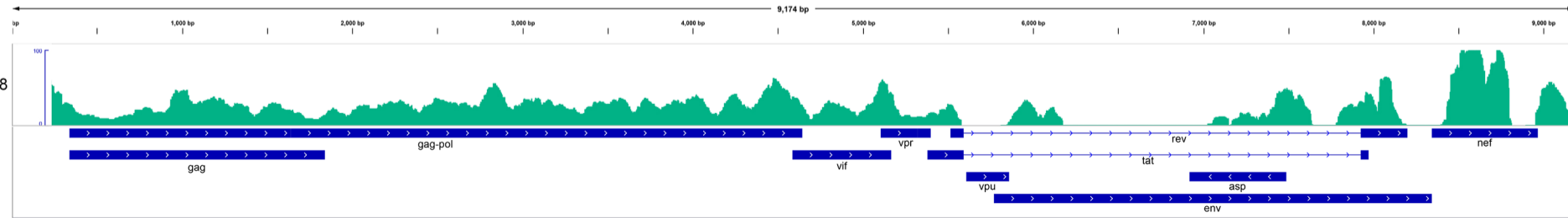

Supplement: FIG S1 [file mSphere.00248-19-sf001.pdf]

log2(TPM + 1)

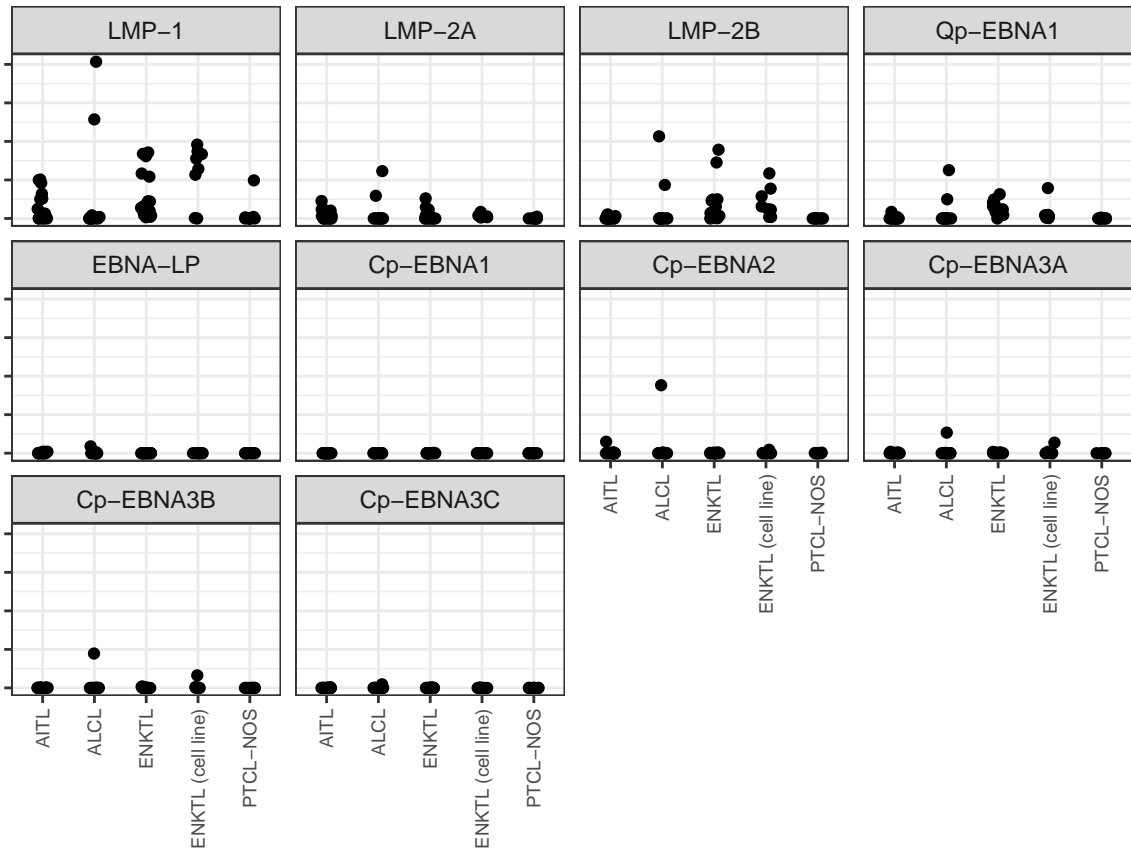

PTCL type

Supplement: FIG S2 [file mSphere.00248-19-sf002.pdf]

CIBERSORT absolute score

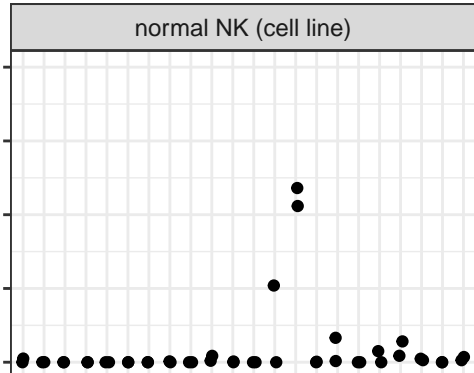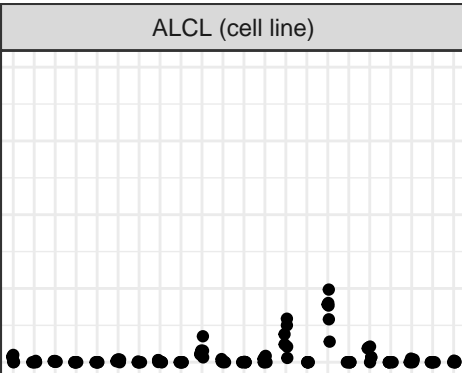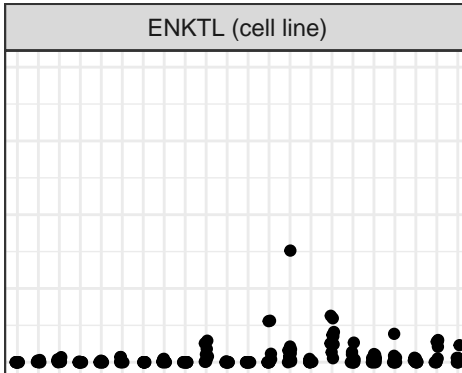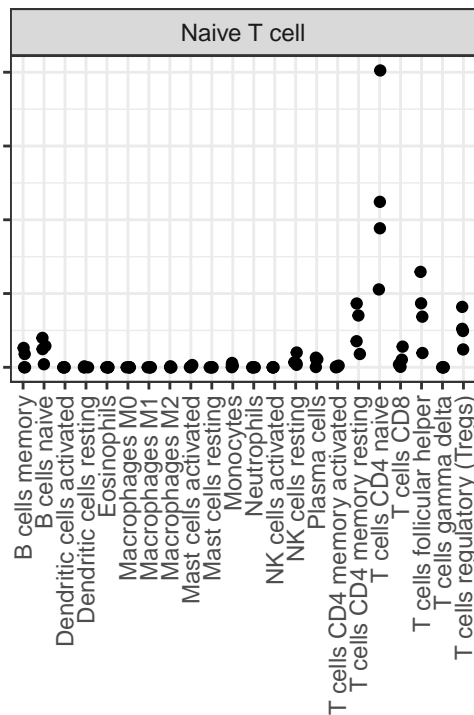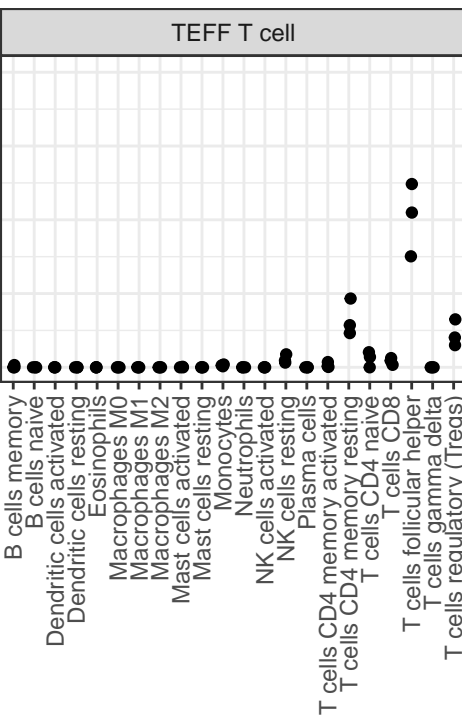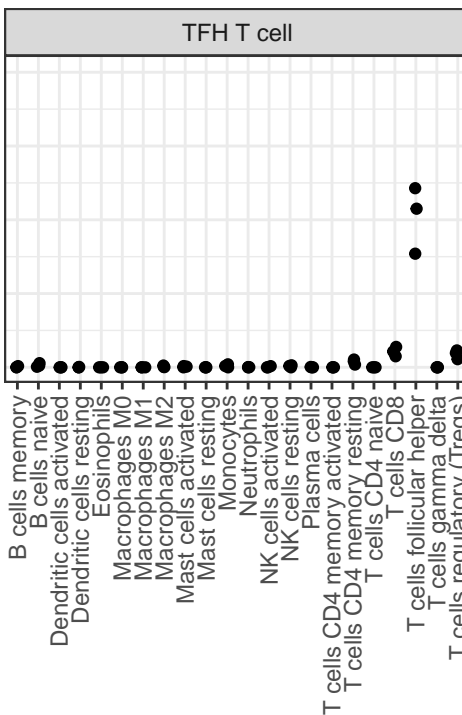

Cell type

Supplement: FIG S3 [file mSphere.00248-19-sf003.pdf]
